# Self‐Reported Sleep Disturbances over the Menopausal Transition and Fracture Risk: The Study of Women's Health Across the Nation

**DOI:** 10.1002/jbm4.10762

**Published:** 2023-05-27

**Authors:** Jane A. Cauley, Howard M. Kravitz, Kristine Ruppert, Yinjuan Lian, Martica J. Hall, Sioban D. Harlow, Joel S. Finkelstein, Gail Greendale

**Affiliations:** ^1^ School of Public Health, Department of Epidemiology University of Pittsburgh Pittsburgh Pennsylvania USA; ^2^ Department of Psychiatry and Behavioral Sciences Rush University Medical Center Chicago Illinois USA; ^3^ Department of Family and Preventive Medicine Rush University Medical Center Chicago Illinois USA; ^4^ Department of Psychiatry University of Pittsburgh Pittsburgh Pennsylvania USA; ^5^ Department of Medicine, Endocrine Unit Massachusetts General Hospital Boston Massachusetts USA; ^6^ David Geffen School of Medicine University of California Los Angeles California USA; ^7^ Department of Epidemiology University of Michigan Ann Arbor Michigan USA

**Keywords:** FRACTURE, GENERAL POPULATION STUDIES, MENOPAUSE, OSTEOPOROSIS, SLEEP, SWAN

## Abstract

Sleep disturbances are common and may impact fracture risk directly by influencing bone turnover or indirectly through shared risk factors or mediators. To investigate the association between self‐reported sleep disturbances across the menopausal transition (MT) and fractures, we prospectively studied 3101 women enrolled in the Study of Women's Health Across the Nation (SWAN). At each of 14 study visits spaced approximately 18 months apart, a standardized validated scale ascertained trouble falling asleep, waking up several times during the night, and waking up earlier than planned. Two time‐varying exposures were modeled: presence of any of the three disturbances at least three times per week and waking up several times during the night at least three times per week. Base models adjusted for fixed (race/ethnicity, study site) and time‐varying characteristics (age, body mass index, and MT stage). Fully adjusted models also included time‐varying bone beneficial and detrimental medications, smoking, alcohol, physical activity, diabetes, depression and sleep medications, and depressive symptoms. Women who experienced a fracture were more likely to report a greater frequency of having trouble falling asleep, waking up several times, and/or waking up earlier: 35% versus 30% at baseline, *p* = 0.02. In the base models, women who had any of the three sleep disturbances at least three times per week had a higher risk of any fracture, odds ratio (OR) = 1.23 (95% confidence intervals, 1.02, 1.48) and nontraumatic fracture, OR = 1.36 (1.03, 1.80). These associations were largely attenuated to nonsignificance in the fully adjusted model. Sensitivity analyses limiting our sample to 2315 SWAN women enrolled in the bone mineral density (BMD) centers yielded similar results. Additional adjustment for femoral neck BMD had no effect on our results. In conclusion, self‐reported sleep disturbances were associated with an increased risk of fractures, but these associations likely reflect shared risk factors or factors in the causal pathway. © 2023 The Authors. *JBMR Plus* published by Wiley Periodicals LLC on behalf of American Society for Bone and Mineral Research.

## Introduction

Osteoporosis is a major public health problem with estimates that one in three women at least 50 years old will experience an osteoporotic fracture in their lifetime.^(^ ^1^ ^)^ Osteoporotic fractures are clearly multifactorial, and many risk factors have been identified.^(^ ^2^ ^)^ Recently, sleep disturbances, also common among women, have emerged as a potentially new modifiable risk factor for fractures. To our knowledge, there have been few prospective studies of sleep and fractures.^(^ ^3^, ^4^, ^5^, ^6^ ^)^ An association between sleep and fractures is biologically plausible. There may be shared risk factors for both sleep disturbances and fracture such as age, weight change, declines in physical activity, poor cognitive and physical function, depression, frailty, and increasing number of comorbidities, including specific comorbidities, for example, diabetes.^(^ ^7^, ^8^, ^9^, ^10^ ^)^ Many of these factors may also be in the causal pathway between sleep and fractures. Sleep disturbances may also be linked to fracture risk through direct mechanisms (e.g., influences on bone turnover, inflammation, vitamin D deficiency, hypogonadism, and insulin resistance).^(^ ^11^ ^)^ Finally, sleep disturbances have been shown to be associated with a higher risk of recurrent falls in postmenopausal women^(^ ^4^ ^)^ and thereby fractures since most fractures occur because of a fall.

Given the paucity of prospective studies of sleep disturbances and fracture, we tested the hypothesis that women with more frequent sleep disturbances over the menopausal transition will have a higher incident clinical fracture risk. These analyses were carried out in the Study of Women's Health Across the Nation (SWAN).

## Methods

**Fig. 1:**
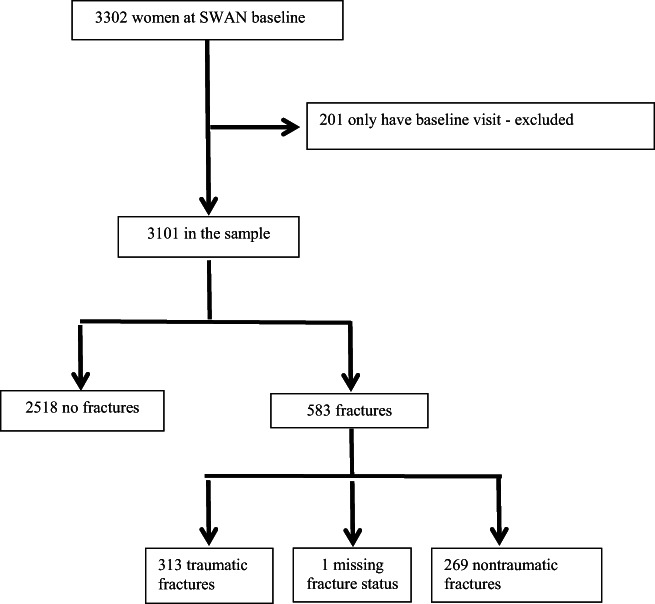
Flowchart for analytical sample.

SWAN is a multisite, longitudinal cohort study of midlife that is being conducted in a community‐based sample of 3302 women at seven clinical sites.^(^ ^12^ ^)^ Enrollees were aged 42 to 52 years with an intact uterus and at least one ovary who were still menstruating 3 months before screening and were not using oral contraceptives or postmenopausal hormone therapy or pregnant/lactating. Each site recruited White women and one racial/ethnic minority. Black women were enrolled at four sites (Boston, MA; Chicago, IL; Detroit, MI; and Pittsburgh, PA); Japanese women were enrolled in Los Angeles, CA; Chinese women were enrolled in Oakland, CA; and Hispanic women were enrolled in New Jersey. A flowchart of the analytic sample is shown in Fig. 1. We excluded 201 women who only had a baseline visit; our final analytic sample included 3101 women. The study was approved by the appropriate committees on human research at each of the seven SWAN clinical sites and the coordinating center, and all women gave written informed consent.

### Sleep assessment

Information on self‐reported sleep disturbances was obtained at baseline and at most study visits through Visit 15 (Visit 00 [baseline] through Follow‐up Visit 10; and Follow‐up Visits 12, 13, and 15). We used a sleep questionnaire that was validated in the Women's Health Initiative.^(^ ^13^, ^14^ ^)^ Information was collected on self‐reported trouble falling asleep (sleep initiation problems), waking up several times per might (sleep maintenance problems), and waking up earlier than planned and unable to fall asleep again (early morning awakening). Frequency of sleep problems in the past 2 weeks were recorded as none, less than one per week, one or two times per week, three or four times per week, and five or more times per week. Sleep disturbances were analyzed as *any* of the three sleep disturbances at least three times per week and individually, waking up several times per night at least three times per week versus less than three times per week since this was the most frequent disturbance reported.

### Bone mineral density

Bone mineral density (BMD) data were collected at five of the seven SWAN sites (Boston, MA; Oakland, CA; Los Angeles, CA; Detroit, MI; and Pittsburgh, PA). At baseline, the BMD cohort consisted of 2335 participants from the five sites. The scans of the femoral neck (FN) were obtained at every visit using a Hologic QDR scanner (Hologic, Inc., Bedford, MA, USA) and the OsteoDyne Hip Positioner System (Osteodyne Inc., Raleigh, NC, USA). While three sites used the 4500A model throughout, two sites upgraded from 2000 to 4500A models starting at Follow‐up Visit 8. A cross‐calibration study of 40 women who were scanned on the old and new machines was carried out to develop calibration regression equations. A quality control program was conducted in collaboration with Synarc, Inc. (San Mateo, CA, USA), through daily phantom measurements, cross‐site calibration every 6 months with an anthropomorphic spine standard, central review of any scans that met the criteria for problem flagging, central review of 5% random sample of scans, and local review of scans at all sites. The short‐term measurement variability in vivo for FN BMD was 0.016 g/cm^2^ (2.2%).

### Fractures

Incident fractures that occurred after baseline were ascertained at each study visit by self‐report up to and including Visit 7. Fractures were adjudicated after Visit 7. The accuracy of the self‐report of fractures was verified by review of radiology reports. The false‐positive rate was 4.6%. We included both traumatic (e.g., due to sports injury, motor vehicular accident) and nontraumatic fractures because both have been linked to low BMD.^(^ ^15^ ^)^ All fractures except digit and facial fractures were included in the analysis.

### Determination of menopause stage

At each visit, menopause transition stage was determined based on reports of the frequency and regularity of menstrual bleeding, as previously described.^(^ ^16^ ^)^ Women were classified as premenopausal if they had experienced at least one menstrual period in the last 3 months with no change in the regularity of their menstrual bleeding during the last year. Women were classified as early perimenopausal if they had experienced at least one menstrual period in the last 3 months with some change in the regularity of their menstrual bleeding during the last year. Women were classified as late perimenopausal if they had experienced no menstrual bleeding in the last 3 months but some menstrual bleeding during the last 11 months. Women were classified as postmenopausal once they had experienced at least 12 consecutive months of amenorrhea. Once a woman had transitioned to a more advanced menopause stage, she could not be reclassified into an earlier stage.

### Other measurements

Baseline and follow‐up questionnaires requested information on age, race/ethnicity, education, reproductive and menstrual history, health status, medication use including sleep and depression medications, and lifestyle factors (alcohol, smoking, and physical activity). Height (centimeters) and weight (kilograms) were measured at each clinic visit and used to calculate the body mass index (BMI) as weight (in kilograms) divided by height squared (meters). Physical activity was measured using the Kaiser Physical Activity Survey (KPAS), which was adapted from the short physical activity survey developed by Baecke et al.^(^ ^17^, ^18^ ^)^ The questionnaire asked for five‐level categorical, Likert‐type responses, ranging from 1 for never to 5 for always for a series of questions about usual level of participation in sports and exercise and active living (defined as walking or biking to work and television watching; the latter is reversed scored). The range in the physical activity scale was 2–10. Alcohol consumption was assessed with a questionnaire or food frequency questionnaire depending on the year of the visit. Total alcohol consumption was classified as none (less than one drink/month), moderate (more than one drink/month, no more than one drink/week), and high (more than one drink/ week). Indicator variables captured the use of medications that tend to reduce fracture risk (“bone beneficial” medications, e.g., systemic hormone use, bisphosphonates, raloxifene, calcitonin, calcitriol, and parathyroid hormone) and medications that tend to increase fracture risk (“bone detrimental,” e.g., corticosteroids, GnRH agonists, aromatase inhibitors, chemotherapy, or antiseizure medications). These medications were entered as time varying, as “any” of these medications at each visit. Depressive symptoms were assessed with the Center for Epidemiologic Studies‐Depression (CES‐D) Scale.^(^ ^19^ ^)^

### Statistical analyses

We used *X* ^2^ and *t* tests to compare baseline characteristics between women who experienced a fracture and women without a fracture. Longitudinal measures of sleep disturbances, were added to discrete survival models as time‐varying exposures. Sleep exposures were censored in the period after the fracture. The odds ratios (OR) and 95% confidence interval (CI) were estimated. Base models adjusted for fixed (race/ethnicity, study site) and time‐varying characteristics (age, body mass index and MT stage). Fully adjusted models included the base model and time‐varying bone beneficial and detrimental medications, smoking, alcohol, physical activity, diabetes, antidepressants, sleep medications, and depressive symptoms. We tested for a time by sleep disturbance interaction in our models. In sensitivity analyses, we limited our sample to 2315 women enrolled in SWAN at the five SWAN BMD sites and further adjusted for FN BMD.

## Results

After an average (SD) follow‐up of 8.8 ± 4.4 years (range 1–15 years), 583 women reported a fracture including 313 (53.8%) traumatic and 269 (46.2%) nontraumatic. Trauma status was missing for one fracture. Women who experienced an incident fracture were more likely to be white and premenopausal (versus early perimenopausal) at baseline, to report a higher education and more frequent alcohol consumption (>1 drink/week), and to have diabetes and take bone detrimental medications (Table 1). There were no statistically significant differences in age, BMI, physical activity, CES‐D score, tobacco use, self‐reported history of osteoporosis, use of supplemental calcium and vitamin D, and bone beneficial medications between women who experienced incident fractures and women who did not fracture. Of the 583 women with incident fracture, 581 had information on their menopausal status at the time of the fracture: 31 (5.3%) surgical menopause, 355 (61.1%) natural postmenopausal, 37 (6.4%) late perimenopausal, 94 (16.2%) early perimenopausal, 22 (3.8%) premenopausal, 24 (4.1%) unknown because of hormone therapy, and 18 (3.1%) unknown because of hysterectomy.

**Table 1:** Baseline Characteristics: No Fractures vs. Fractures Mean (SD) or *n* (%)

Variable	Total *N* = 3101	No fracture *N* = 2518	Fracture *N* = 583	*p* value^a^
Age (years), mean (SD)	46.4 (2.7)	46.3 (2.7)	46.5 (2.7)	0.29
Body mass index (kg/m^2^), mean (SD)	28.2 (7.2)	28.1 (7.2)	28.4 (7.5)	0.54
CES‐D score, mean (SD)	10.6 (9.6)	10.6 (9.6)	10.4 (9.5)	0.85
Race/ethnicity, *n* (%)				<.0001
White	1470 (47.4)	1129 (44.8)	341 (58.5)	
Black	882 (28.4)	737 (29.3)	145 (24.8)	
Chinese	245 (7.9)	206 (8.2)	39 (6.7)	
Hispanic	229 (7.4)	207 (8.2)	22 (3.8)	
Japanese	275 (8.9)	239 (9.5)	36 (6.2)	
Educational attainment, *n* (%)				0.03
≤ high school	731 (23.8)	613 (24.6)	118 (20.3)	
Tobacco use, *n* (%)				0.66
Past	794 (25.6)	641 (25.5)	153 (26.2)	
Current	517 (16.7)	427 (17.0)	90 (15.4)	
Alcohol use, *n* (%)				0.015
None (<1 drink/month)	1531 (49.5)	1270 (50.6)	261 (44.9)	
Moderate use (>1 drink/month)	885 (28.6)	714 (28.5)	171 (29.4)	
High use (>1 drink/week)	675 (21.8)	525 (20.9)	150 (25.8)	
Physical activity, mean (SD)	7.7 (1.8)	7.7 (1.8)	7.7 (1.8)	0.34
Preexisting conditions, *n* (%)				
Diabetes	139 (4.5)	100 (4.0)	39 (6.7)	0.004
Arthritis	515 (16.8)	404 (16.2)	111 (19.2)	0.09
Osteoporosis	36 (1.2)	27 (1.1)	9 (1.6)	0.34
Menopausal status, *n* (%)				0.05
Pre‐	1411 (45.8)	1123 (44.9)	288 (49.7)	
Early Peri‐	1667 (54.1)	1377 (55.0)	290 (50.0)	
Unknown	5 (0.16)	3 (0.12)	2 (0.34)	
Supplemental calcium, *n* (%)				0.08
Yes	1435 (46.3)	1146 (45.5)	289 (49.6)	
Supplemental vitamin D, *n* (%)				0.15
Yes	1164 (37.5)	930 (36.9)	234 (40.1)	
Bone medication use^b^, *n* (%)				0.75
Beneficial	3 (0.1)	1 (0.04)	2 (0.34)	0.09
Detrimental	98 (3.2)	70 (2.8)	28 (4.8)	0.01
Depression/sleep medications, *n* (%)				
Yes	245 (7.9)	194 (6.3)	51 (8.8)	0.40
Cause of fracture, *n* (%)				
Traumatic	‐	‐	313 (53.8)	
Nontraumatic	‐	‐	269 (46.2)	
Missing			1	
Sleep disturbances^c^, *n* (%)				
Trouble falling asleep	316 (10.2)	239 (9.5)	77 (13.25)	0.008
Waking up several times	805 (26.1)	632 (25.2)	173 (29.8)	0.02
Waking up earlier	387 (12.5)	301 (12.0)	86 (14.8)	0.07
Any of the three sleep disturbances, *n* (%)	956 (31.0)	752 (30.0)	204 (35.1)	0.02

^a^ *p* value is testing difference in means (continuous variables) or proportions (categorical variables) between those with and without fracture.

^b^ Bone beneficial medications include systemic hormone use, bisphosphonates, raloxifene, calcitonin, calcitriol, and parathyroid hormone); bone detrimental medications include corticosteroids, GnRH agonists, aromatase inhibitors, chemotherapy, or antiseizure medications. Use refers to any use of a medication.

^c^ Sleep disturbances reported >3 times per week.

**Fig. 2:**
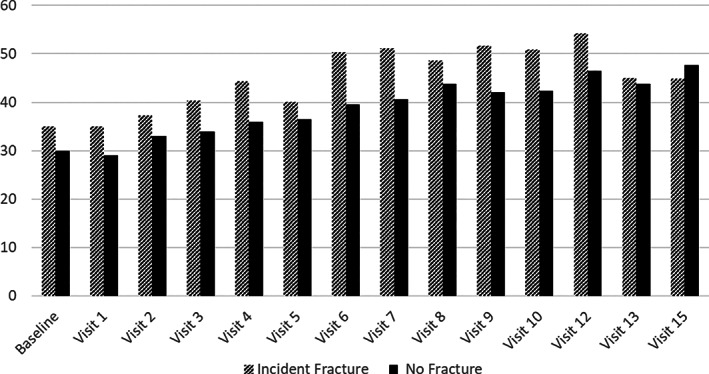
Prevalence of sleep disturbances (three or more times per week) across study visits: stratified by incident fracture status.

At baseline, women who subsequently experienced a fracture were more likely to report a greater frequency of trouble falling asleep, waking up several times, and waking up earlier than planned prior to the event. About 35% of women who had a fracture reported one or more of the three sleep disturbances, compared to 30% of women who did not (*p* = 0.02). Over time, the percentage of women who reported three or more sleep disturbances per week increased but was generally higher in those who fractured (Figure 2).

### All fractures

In the base models, women who had one or more of the three sleep disturbances had a 23% (OR = 1.23; 95% CI: 1.02 to 1.48) higher risk of any fracture (Table 2). This association was attenuated to 18% (OR = 1.18; 95% CI: 0.96 to 1.45) higher risk in the fully adjusted multivariable (MV) model and was no longer statistically significant. Similar findings were obtained for the specific sleep disturbance of waking up several times per night at least three times per week over the previous 2 weeks with a 24% (OR = 1.24; 95% CI: 1.03 to 1.50) and 17% (OR = 1.17; 95% CI: 0.95 to 1.43) higher risk of any fracture in the base and fully adjusted models, respectively. There was no evidence of an interaction between time and sleep disturbance.

**Table 2:** Odds Ratio (OR) (95% Confidence Interval [CI]) of Fracture by Sleep Disturbances

Models	Any of the three sleep disturbances ≥3 times per week OR (95% CI)	Waking up several times per night ≥3 times per week OR (95% CI)
All fractures (*N* = 3101)		
Base model	1.23 (1.02, 1.48)	1.24 (1.03, 1.50)
Full MV model	1.18 (0.96, 1.45)	1.17 (0.95, 1.43)
Non‐traumatic fractures (*N* = 2787)		
Base model	1.36 (1.03, 1.80)	1.45 (1.09, 1.91)
Full MV model	1.33 (0.98, 1.82)	1.27 (1.01, 1.87)

*Note*: Base model = age, race/ethnicity, site, body mass index, menopausal transition stage.

*Note*: Full MV model = base + bone beneficial and bone detrimental medications, smoking, alcohol, physical activity, diabetes, depression and sleep medications and depressive symptoms status.

Abbreviation: MV = Multivariable.

### Nontraumatic fractures

Examination of nontraumatic fractures showed stronger relationships with sleep disturbances (Table 2). Women who reported any of the three sleep disturbances at least three times per week had a 36%  (OR = 1.36; 95% CI: 1.03 to 1.80) increased risk of fractures in the base model. Women who reported waking up several times had a 45%   (OR = 1.45; 95% CI: 1.09 to 1.91) increased risk of fracture in the base models. The association with 3 or more sleep disturbances at least three times per week was attenuated and was no longer significant in the fully adjusted models: OR = 0.33 (95% CI 0.98 to 1.82). However, the association with waking up several times per night and non‐traumatic fractures remained significant in the fully adjusted model (OR = 1.27; 95% CI 1.01 to 1.87).

### Sensitivity analyses

In sensitivity analyses limiting our sample to women enrolled in the BMD sites, the results were very similar to those in the full SWAN cohort (Table 3). The significant association between at least three sleep disturbances per week or waking up several times per night at least three times per week and both all fractures and nontraumatic fractures was attenuated in the full MV model. Additional adjustment for FN BMD had no effect on our results.

**Table 3:** Odds Ratio (OR) (95% Confidence Interval [CI]) of Fracture by Sleep Disturbance: Sensitivity Analysis Limited to Women Enrolled in SWAN at the Five Bone Mineral Density Centers

Models	Any of the three sleep disturbances ≥3 times per week OR (95% CI)	Waking up several times per night ≥3 times per week OR (95% CI)
All fractures (*N* = 2315)		
Base model	1.24 (1.02, 1.53)	1.22 (1.00, 1.51)
Full MV model	1.17 (0.94, 1.47)	1.13 (0.90, 1.41)
Full MV model + femoral neck BMD	1.18 (0.93, 1.49)	1.15 (0.91, 1.45)
Nontraumatic fractures (*N* = 2060)		
Base model	1.44 (1.06, 1.95)	1.48 (1.09, 2.01)
Full MV model	1.33 (0.95, 1.87)	1.33 (0.95, 1.86)
Full MV model + femoral neck BMD	1.33 (0.93, 1.89)	1.39 (0.98, 1.97)

*Note*: Base model = age, race/ethnicity, site, body mass index, menopausal transition stage.

*Note*: Full MV model = base + bone beneficial and bone detrimental medications, smoking, alcohol, physical activity, diabetes, depression and sleep medications and depressive symptoms status.

Abbreviation: MV = multivariable.

## Discussion

This study prospectively examined the relationship between sleep disturbance and fracture risk in midlife women. We found that women who reported any sleep disturbance at least three times per week, considered a clinically relevant frequency of self‐reported sleep problems^(^ ^20^, ^21^ ^)^ had a 23% increased risk of any fracture and a 36% increased risk of a nontraumatic fracture compared to women who reported sleep disturbances less frequently. The greater association between sleep and nontraumatic fractures may suggest a link between the low bone mass or bone turnover and sleep disturbances. However, these associations between sleep disturbance and fracture were attenuated to nonsignificance after adjusting for characteristics that may reflect shared risk factors or factors that are in a physiological pathway linking sleep disturbance to fracture. For example, we showed in a much older cohort of women, the Study of Osteoporotic Fractures (SOF), that an aggregate measure of self‐reported sleep health as defined by satisfaction with sleep duration, daytime sleepiness, midsleep time, sleep latency, and sleep duration was associated with prevalent and incident depression,^(^ ^22^ ^)^ and depression has also been linked to fractures.^(^ ^23^ ^)^ Similarly, sleep disorders are highly prevalent in people with type 2 diabetes,^(^ ^24^ ^)^ and diabetes has been associated with an increased risk of fracture.^(^ ^25^ ^)^ Menopausal status has been shown to influence the incidence of obstructive sleep apnea (OSA), a condition that is associated with sleep disturbances, most notably with comorbid insomnia.^(^ ^26^, ^27^ ^)^ In the Nurses' Health Study, incident OSA was associated with the type of menopause (surgical/natural) and earlier age at menopause. Menopausal status also influences rates of bone loss and fracture. We have shown in SWAN that bone loss accelerates during the transmenopausal period,^(^ ^28^ ^)^ which could lead to an increased risk of fracture. Thus, poor sleep may be a marker of these underlying characteristics and conditions that are associated with fractures or could be in the biological pathway linking poor sleep to fractures. What is important is that adjustment for these covariates attenuated the sleep–fracture associations.

Low BMD is an important risk factor for fracture.^(^ ^29^ ^)^ Self‐reported short sleep duration has been associated with low BMD in the women enrolled in the Women's Health Initiative.^(^ ^30^ ^)^ In the SOF study, short sleep measured by actigraphy was associated with *higher* total hip BMD in unadjusted analyses. However, after adjusting for BMI, the association was attenuated to nonsignificance.^(^ ^31^ ^)^ In our sensitivity analysis, further adjusting for FN BMD had no effect on our results.

As noted earlier, the prevalence of self‐reported sleep disorders at baseline was high, with about a third of all women reporting trouble falling asleep, waking up several times per night, or waking up earlier than planned and unable to fall back asleep. The prevalence increased over time, such that by later follow‐up visits, approximately 40% to 50% of women reported three or more sleep disturbances per week. This high prevalence highlights efforts that are needed to identify those with specific treatable sleep problems.

To our knowledge, few prospective studies have examined sleep and clinical outcomes of fractures in women. In the Women's Health Initiative, with a mean age of 63, the investigators found a modest (10% to 13%) increased risk of all fractures, upper limb, lower limb, and central fractures among short sleepers, but there was no association with long sleep.^(^ ^4^ ^)^ There was modest evidence of an association between other sleep characteristics (insomnia, sleep quality, sleep disturbance level) and fractures. In SOF, a much older (mean age 77 years) cohort of women, women who reported napping daily had a 33% increased risk of hip fracture; long sleep duration (>9 h) was associated with an increased risk of both hip and nonspine fractures.^(^ ^5^ ^)^ In the Nurses' Health Study, self‐reported OSA was associated with a twofold increased risk of vertebral fractures but not with hip fractures.^(^ ^3^ ^)^ Most of these studies were carried out in much older women and are not directly comparable to women enrolled in SWAN. Our study is the first study of sleep and fractures in women at midlife.

The major limitation to all these prospective studies of sleep was their reliance on self‐reporting. There is poor agreement between self‐reporting and objective measures of sleep. For example, in both SOF and the Osteoporotic Fractures in Men Sleep Study, the agreement between self‐reported sleep duration and actigraphic sleep duration was poor (kappa = 0.24).^(^ ^6^ ^)^ In SWAN, however, sleep parameters were comparable on the basis of both actigraphy and polysomnography (PSG), but there was little agreement with sleep diaries; estimates of sleep duration were higher in diaries compared to both actigraphy and PSG.^(^ ^32^ ^)^ Nevertheless, self‐report/sleep diaries are the clinical “gold standard” for assessing insomnia complaints and can be obtained concurrently with noninvasive actigraphy. Information on both can be included in studies relatively inexpensively with wearable devices. In addition, the sleep‐disturbance questions that we used were from a validated questionnaire.^(^ ^13^, ^14^ ^)^ Our three items were developed in a multiracial/ethnic sample of postmenopausal women aged 50–79, and we have administered them repeatedly since the SWAN baseline assessment in 1996–1997.^(^ ^33^ ^)^

Ideally, when examining sleep and health outcomes, self‐reported sleep disturbances should be objectively confirmed. However, in a recent review, Cudney et al. commented that their findings suggested that self‐report remains the most consistent way of measuring sleep quality since it is unclear which markers of objective sleep are the most consistent.^(^ ^34^ ^)^ Finally, our study was observational, and residual confounding by other unmeasured factors is an inherent limitation of this type of study.

However, this study has a number of strengths. We collected information on sleep disturbances over the menopausal transitions and prospectively linked them to fractures in a cohort of women who were followed up to 15 years. We adjusted for important covariates including both fixed and time‐varying risk factors.

In conclusion, about a third of midlife women self‐reported sleep disturbances before menopause, and the prevalence increased over time. Self‐reported sleep disturbances were associated with an increased risk of all fractures and more strongly, though nonstatistically significant, in the MV model for nontraumatic fractures. These associations were attenuated after adjusting for shared risk factors and factors that could be in the physiological pathway between sleep and fractures.

## Author Contributions

**Jane A. Cauley:** Conceptualization; data curation; writing – original draft; writing – review and editing. **Howard Kravitz:** Writing – review and editing. **Kristine Ruppert:** Data curation; writing – review and editing. **Yinjuan Lian:** Data curation; writing – review and editing. **Martica J. Hall:** Writing – review and editing. **Sioban D. Harlow:** Writing – review and editing. **Joel Finkelstein:** Writing – review and editing. **Gail Greendale:** Writing – review and editing.

## Conflict of Interest

JAC, HMK, KR, YL, MH, SDH, JSF, and have no conflicts.

### Peer Review

The peer review history for this article is available at https://www.webofscience.com/api/gateway/wos/peer-review/10.1002/jbm4.10762.
